# Integrating isothermal amplification with magnetic flocculation and DNA-guided PfAgo for SARS-CoV-2 detection

**DOI:** 10.3389/fmicb.2026.1792924

**Published:** 2026-04-08

**Authors:** Xiaoxing Zhou, Mengru Xie, Xia Yu, Leping Ning, Chao Ye, Yilian Zhao, Yan Wei, Xinchu Liu, Jinghui Ma, Jilin Qing, Zhencheng Chen, Zhizhong Chen

**Affiliations:** 1 School of Clinical Medicine, Guilin Medical University, Guilin, Guangxi, China; 2 The First Clinical Medical College, Guangxi Medicine University, Nanning, Guangxi, China; 3 Joint Inspection Center of Precision Medicine, The People’s Hospital of Guangxi Zhuangzu Autonomous Region and Guangxi Academic of Medical Sciences, Nanning, Guangxi, China; 4 Clinical Laboratory, The People’s Hospital of Guangxi Zhuangzu Autonomous Region and Guangxi Academic of Medical Sciences, Nanning, Guangxi, China; 5 Clinical Laboratory, The People’s Hospital of Fengjie, Chongqing, China; 6 Center for Reproductive Medicine and Genetics, The People’s Hospital of Guangxi Zhuangzu Autonomous Region and Guangxi Academic of Medical Sciences, Nanning, Guangxi, China; 7 School of Electronic Engineering and Automation, Guilin University of Electronic Technology, Guilin, Guangxi, China

**Keywords:** diagnosis, flocculation, pathogen detection, PfAgo protein-based detection, SARS-CoV-2

## Abstract

**Introduction:**

The recurrent threat of respiratory infectious disease outbreaks creates an urgent need for diagnostic tests that are rapid, portable, and user-friendly, applicable across diverse settings—from central laboratories and hospitals to field deployments, resource-limited regions, and home use.

**Methods:**

In response, we developed three novel nucleic acid detection platforms—RT-RAA-MPs, RT-RAA-F-PfAgo, and RT-RAA-L-PfAgo—that integrate reverse transcription-recombinase-aided amplification (RT-RAA) with distinct instrument-free readout mechanisms. These systems utilize custom-designed primers and guide DNA (gDNA) targeting the SARS-CoV-2 N and ORF genes.

**Results:**

Performance evaluation demonstrated high analytical sensitivity, with detection limits of 10^2^ copies/μL for RT-RAA-MPs and 5 copies/μL for both RT-RAA-F-PfAgo and RT-RAA-L-PfAgo. All assays exhibited excellent specificity, showing no cross-reactivity with nine common respiratory pathogens. Clinical validation using 23 patient samples revealed perfect agreement with RT-qPCR (100% concordance), supported by a Kappa value of 1.00, indicating outstanding diagnostic consistency and statistical robustness.

**Discussion:**

With lyophilized reagent compatibility and minimal equipment requirements, these platforms are readily adaptable for a wide range of applications, including clinical diagnosis at all hospital levels, point-of-care screening, border control inspection, and environmental monitoring. This work establishes a versatile, reliable, and deployable toolkit for effective outbreak response and decentralized molecular diagnostics.

## Graphical abstract

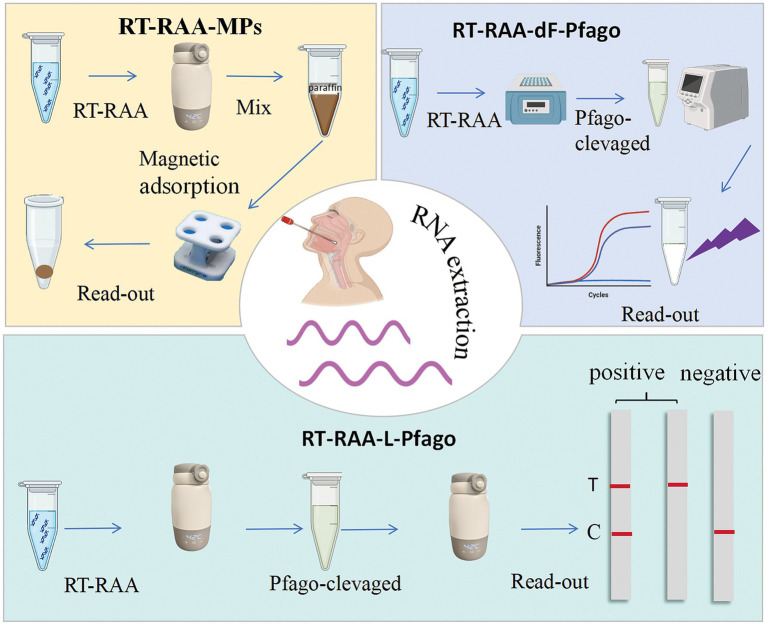

## 1 Introduction

SARS-CoV-2 is a respiratory pathogen characterized by rapid transmission, strong infective, and high mutation rates (Contini et al., 2023). Since its emergence in late 2019, it has caused a global pandemic with severe impacts on worldwide health, society, and economy (Wang and Naeem, 2025). Although the World Health Organization declared the end of the SARS-Cov-2 Public Health Emergency of International Concern (PHEIC) in May 2023, SARS-CoV-2 continues to circulate at low levels globally with periodic resurgences (Carvajal et al., 2024). The SARS-CoV-2 pandemic has underscored an urgent need for diagnostic technologies that are not only accurate but also rapid, accessible, and deplorable in diverse settings to enable effective outbreak control. While the timely detection of respiratory pathogens remains a cornerstone of epidemic containment, conventional diagnostic platforms face significant practical constraints. Gold-standard methods such as RT-qPCR, despite high sensitivity and specificity, rely heavily on expensive instrumentation, specialized laboratory infrastructure, and trained operators (Gupta et al., 2021; Hernández-Huerta et al., 2021). Digital PCR (ddPCR) enables absolute quantification of nucleic acids through reaction partitioning and demonstrates higher sensitivity and reproducibility than RT-qPCR in low viral load samples without the need for standard curves (Wang et al., 2025). However, its application in point-of-care testing (POCT) remains limited. Similarly, next-generation sequencing (Hu et al., 2021) offers unparalleled pathogen identification capability but is impeded by high costs, complex workflows, and substantial bioinformatics demands, rendering it unsuitable for rapid field deployment or routine clinical use. These limitations highlight the pressing demand for simpler, more accessible diagnostic alternatives. This study establishes a detection platform using SARS-CoV-2 as a model pathogen. The platform methodology developed here is not only applicable to SARS-CoV-2, but can also be readily adapted for detecting other pathogens through redesign of primers and guide DNAs, thereby extending to emerging respiratory threats and providing a transferable technical framework for future public health preparedness.

Isothermal amplification techniques represent a paradigm shift in nucleic acid testing, offering rapid detection and minimal equipment requirements. Among these, recombinase polymerase amplification (RPA) and recombinase-aided amplification (RAA) have gained prominence for their ability to achieve high-efficiency amplification at constant low temperatures (37–42 °C) within 20–30 min (Diagne et al., 2020). However, current RPA/RAA detection formats present notable limitations that restrict their widespread adoption (Zhao et al., 2024b): fluorescence-based readouts require dedicated instrumentation unavailable in resource-limited settings, while lateral flow assays incur substantial costs from specialized test strips and still involve multiple handling steps (Munawar, 2022). Moreover, the operational complexity and recurring expenses associated with these detection methods render them unsuitable for home testing or deployment in poorly equipped areas. These constraints underscore the urgent need for instrument-independent detection strategies that maintain analytical robustness while eliminating specialized equipment requirements.

In this context, the integration of magnetic bead-based flocculation (MPs) with RPA/RAA systems offers a promising direction toward truly accessible testing. Originally explored in environmental monitoring (Wei et al., 2018), this approach leverages colloidal aggregation to enable visual detection without specialized equipment (Achary et al., 2025). The MPs-based system presents a particularly attractive solution for home use and resource-limited settings, as it eliminates the need for expensive instruments or costly test strips. However, conventional MPs detection still requires post-amplification handling of RPA/RAA products—a process that introduces significant contamination risks and operational challenges (Wee et al., 2015). The adaptation of this methodology to a truly closed-tube format thus represents a crucial step toward realizing equipment-minimized, contamination-resistant detection for decentralized settings.

Beyond contamination control, achieving high detection specificity remains a persistent challenge. The DNA-guided Argonaute protein from *Pyrococcus furiosus* (PfAgo) presents an innovative solution (Xu and Wu, 2026), enabling precise target recognition and cleavage without the PAM sequence restrictions of CRISPR-based systems (van Dongen et al., 2020). PfAgo’s programmable gDNA guidance allows specific discrimination of amplification products, while its thermostable nature facilitates flexible system integration. Although successfully applied in other pathogen detection contexts (Wang et al., 2021; Dai et al., 2025; Xun et al., 2021), its potential for enhancing SARS-CoV-2 detection specificity within equipment-minimized RAA platforms remains largely unexplored. This gap presents an opportunity to develop a new generation of highly specific, instrument-independent detection systems.

This study addresses these challenges through two innovatively engineered RAA-based platforms for SARS-CoV-2 detection. A one-pot RT-RAA-MPs system was developed to eliminate post-amplification handling, integrating amplification and detection within a single sealed tube to prevent contamination while maintaining visual readout capability—making it ideally suited for home testing and resource-limited environments. Environmental simulation and performance validation demonstrate robust functionality across diverse scenarios—from home testing and resource-limited field applications to primary care settings and higher-tier medical facilities. Complementarily, an RT-RAA-PfAgo platform was established to enhance detection specificity, supporting both lateral flow and fluorescence readouts for flexible application across different settings. Collectively, these systems provide a comprehensive, scenario-adaptive diagnostic framework that significantly advances our capacity for responsive outbreak management.

## 2 Materials and methods

This study developed three SARS-CoV-2 detection platforms based on RT-RAA: (1) RT-RAA-MPs (RT-RAA combine Magnetic Particles): An integrated magnetic bead flocculation system enabling visual readout through pH-induced magnetic bead-DNA flocculation without any instrumentation. (2) RT-RAA-F-PfAgo (Single-target fluorescence system): Utilizes PfAgo protein-mediated fluorescent probe cleavage coupled with qPCR instruments for highly sensitive single-gene detection. (3) RT-RAA-dF-PfAgo (Dual-target fluorescence system): An upgraded version of the single-target system enabling simultaneous detection of ORF and N genes. (4) RT-RAA-L-PfAgo (Lateral flow system): Combines PfAgo cleavage with lateral flow strips for visual readout.

### 2.1 Pseudovirus, primers, and reagents

The SARS-CoV-2 pseudovirus reagent was purchased from Sangon Biotech (China). All primers, gDNA, and probes used in the study were synthesized by the same supplier from Sangon Biotech (China). RT-RAA reagents were obtained from Zongce Biotechnology Co., Ltd. (China); magnetic beads were sourced from Thermo Fisher Scientific (United States); T4 polynucleotide kinase was purchased from NEB (United States); and PfAgo protein, corresponding buffer, and Mn^2+^ solution were acquired from tiosbio (China). This selection ensured the consistency and quality of core reaction components.

### 2.2 Nucleic acid extraction

Efficient release of viral RNA is critical for downstream molecular detection. RNA was extracted using a nucleic acid releasing agent: pseudovirus was mixed with the releasing agent at a 5:1 (v/v) ratio and heated at 95 °C for 5 min. The released RNA was then stored at −80 °C until further use. This rapid and equipment-free extraction method facilitates application in resource-limited settings.

### 2.3 Design of primers, gDNAs, and probes

Specific and efficient recognition of viral targets requires carefully designed primers, gDNA, and probes. In this study, primers and gDNA were designed based on the SARS-CoV-2 ORF and N gene sequences (GenBank NC_045512.2). All designs were performed using SnapGene software (GSL Biotech, United States). The RT-RAA primers were 29-bp oligonucleotides, screened and confirmed by 2% agarose gel electrophoresis. The gDNA sequences, 16 bp in length, were phosphorylated using T4 polynucleotide kinase (T4 PNK) and purified by 18% Urea-PAGE prior to use. Probe sequences were designed according to gDNA cleavage sites, targeting the N and ORF genes, designated as N-P and ORF-P, respectively. For fluorescence detection, the 5′ and 3′ ends of N-P-F were labeled with FAM and BHQ1, while ORF-P-F was labeled with ROX and BHQ2. For lateral flow detection, both N-P-L and ORF-P-L were labeled with biotin and FAM at their 5′ and 3′ ends, respectively, as summarized in Table 1.

**Table 1:** Primers, gDNA, and probe sequences for the SARS-CoV-2 ORF and N genes.

Primer	Sequence (5′-3′)
N-F	TCAAGAAATTCAACTCCAGGCAGCAGTAG
N-R	AGTTTGGCCTTGTTGTTGTTGGCCTTTAC
ORF-F	CCGTTGCCACATAGATCATCCAAATCCTA
ORF-R	GTATACGACATCAGTACTAGTGCCTGTGC
gDNA	
N-gDNA 1	AGCAAGAGCAGCATCA
N-gDNA 2	TGTCAAGCAGCAGCAA
ORF-gDNA 1	CATAACCTTTCCACAT
ORF-gDNA 2	TGATCACAACTACAGC
Probe	
N-P-L	FAM-CGCACCCAGCAAAGCAAGAGCAGGTGCG-Biotin
N-P-F	FAM-CGCACCCAGCAAAGCAAGAGCAGGTGCG-BHQ1
ORF-P-L	FAM-GCCACGTACAGCCATAACCTTTCGTGGC-Biotin
ORF-P-F	ROX-GCCACGTACAGCCATAACCTTTCGTGGC-BHQ2

### 2.4 Establishment of the RT-RAA-MPs detection system

We aimed to develop a rapid and equipment-minimized nucleic acid detection method based on magnetic flocculation. The RT-RAA-MPs buffer was prepared by mixing magnetic beads, 4% (W/V) PEG 8000, and nuclease-free water, with pH adjusted to 4.5 using acetic acid, and stored at 4 °C. The amplification system was assembled as follows: 25 μL of A buffer, 1.5 μL of B buffer, 2 μL each of forward and reverse primers for ORF/N genes, 1 μL of RNA template, and nuclease-free water to 50 μL total volume. After incubation at 42 °C for 30 min, 500 μL of RT-RAA-MPs buffer was added and allowed to stand for 5 min. The tube was placed on a magnetic stand for 5 min; results were interpreted based on magnetic bead flocculation. Key parameters—including B buffer volume, amplification temperature and time, pH, and magnetic bead ratio—were optimized via one-factor-at-a-time experiments. Sensitivity was assessed using serial pseudovirus dilutions (10^6^–1 copies/μL), specificity against nine common respiratory viruses, and clinical performance using 23 SARS-CoV-2 positive samples compared to RT-qPCR.

### 2.5 Establishment of the RT-RAA-F-PfAgo detection system

To enable specific and sensitive fluorescence-based detection, we established a PfAgo-mediated cleavage assay. The cleavage reaction consisted of 2 μL PfAgo protein, 2 μL buffer, 1 μL Mn^2+^, and 2 μL ORF-gDNA or N-gDNA. Then, 4 μL of ORF or N gene amplification product was mixed with 5 μL of corresponding fluorescent probe (ORF-P-F or N-P-F), and nuclease-free water was added to 25 μL. After brief centrifugation, the tube was incubated in a qPCR instrument at 95 °C for 30 min, with fluorescence signals collected every 30 s. Results were also observed under 490–495 nm UV light. Parameters including PfAgo, Mn^2+^, cleavage time and temperature were systematically optimized. To improve specificity, a single-tube dual-target system (RT-RAA-dF-PfAgo) was developed, allowing simultaneous amplification of ORF and N genes. Here, 8 μL of purified amplification product was mixed with 5 μL each of ORF-P-F and N-P-F probes, and water to 50 μL, followed by reaction at 95 °C for 30 min with fluorescence monitoring. Probe concentrations for dual-target detection were further optimized. Sensitivity, specificity, and clinical validation were performed as described above.

### 2.6 Establishment of the RT-RAA-L-PfAgo detection system

An LFA-based detection method was established. Based on the RT-RAA-F-PfAgo system, the cleavage reaction incorporated ORF or N gene amplification products and their corresponding lateral flow probes (ORF-P-L or N-P-L). After brief centrifugation, the mixture was heated at 95 °C for 30 min. Then, 5 μL of cleavage product was diluted with 45 μL nuclease-free water, applied to a test strip, and read within 5–10 min. Sensitivity, specificity, and clinical performance were evaluated as in previous systems.

### 2.7 Clinical sample collection

This study was approved by the Ethics Committee of the People’s Hospital of Guangxi Zhuangzu Autonomous Region (IIT-2025-94). Clinical samples included 23 outpatient samples and 17 respiratory infection patient samples. All samples were previously characterized using commercial kits: the 23 throat swabs were tested with SARS-Cov-2 (2019-nCoV) ORF1ab/N Gene Nucleic Acid Detection Kit (Xi’an Tianlong Technology Co., Ltd., China), identifying 18 positives and 5 negatives; the remaining 17 samples were analyzed using a 13 Respiratory Pathogens Multiplex Detection Kit (Ningbo Health Gene Technologies), showing infections with Flu A (4), Flu B (3), HPIV (2), HMPV (2), HADV (2), HRV (3), and *Chlamydia pneumoniae* (1).

## 3 Results

### 3.1 Selection of primers and gDNA

**Figure 1:**
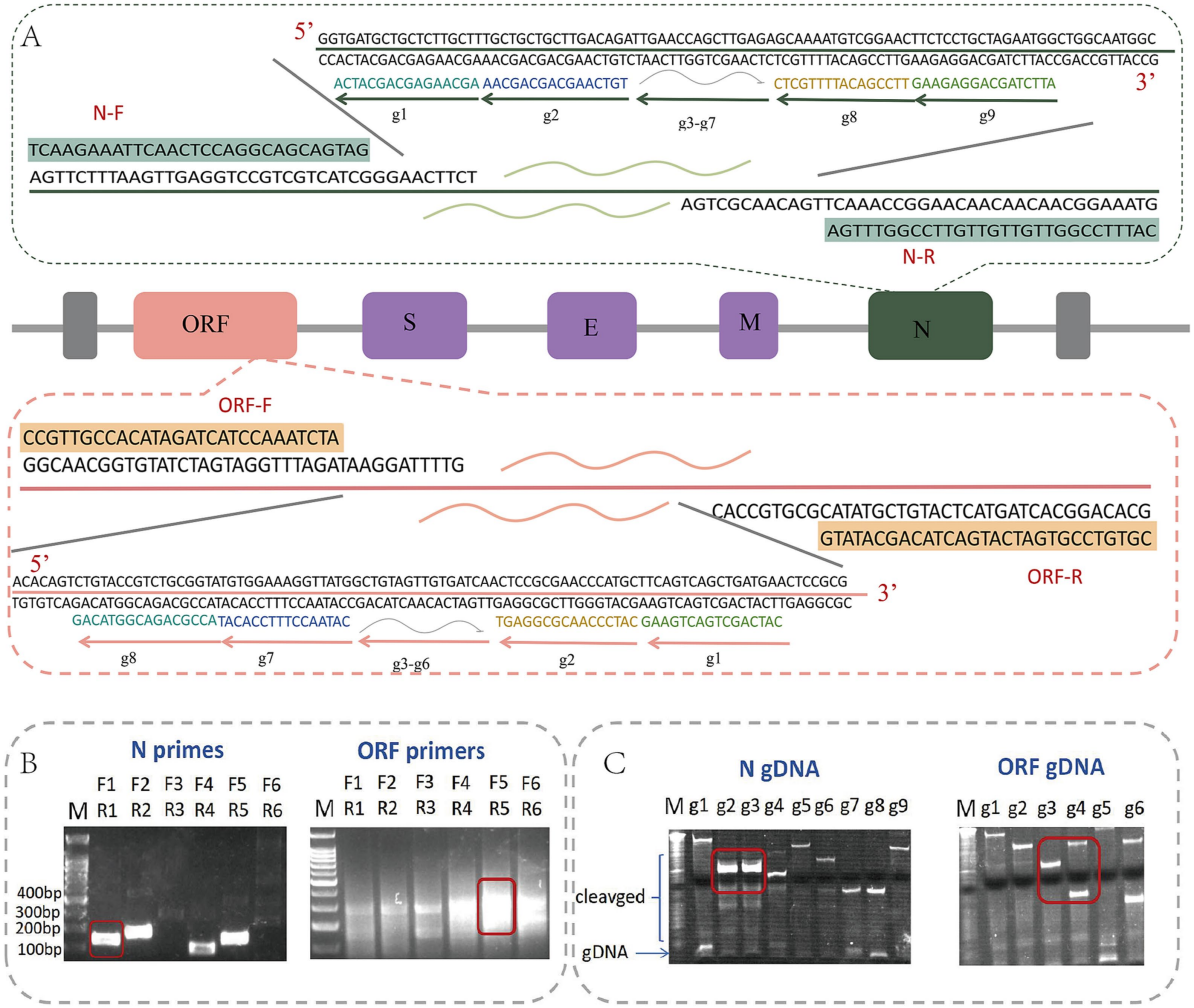
Primer and gDNA design and screening. **(A)** Schematic diagram of primer and gDNA design. **(B)** Results of agarose gel electrophoresis screening for the N gene primer and ORF gene primer. **(C)** Results of 18% Urea-Page screening for N and ORF gDNA.

To establish a robust RT-RAA detection system, we designed and screened primers and gDNA targeting the N and ORF genes of SARS-CoV-2 (Figure 1A). Six primer pairs each for the N and ORF genes were evaluated. Based on amplification efficiency and specificity, N-F1/R1 (158 bp) and ORF-F5/R5 (278 bp) were selected, both yielding single bright bands without non-specific amplification (Figure 1B). Subsequently, a series of gDNA sequences (N-g1–g9 and ORF-g1–g6) were designed along the amplicons and tested for their ability to guide PfAgo-mediated cleavage via 18% Urea-PAGE. Effective cleavage was indicated by disappearance of the target band (Figure 1C). To ensure reliability, two adjacent gDNAs were required to mediate cleavage efficiently; N-g2/g3 and ORF-g3/g4 were ultimately chosen. Stem-loop probes were then designed complementary to the cleavage sites to ensure detection sensitivity and specificity. These results confirm the successful selection of specific primers and gDNA for subsequent assays.

### 3.2 Establishment and performance evaluation of the RT-RAA-MPs

#### 3.2.1 Principle of RT-RAA-MPs

**Figure 2:**
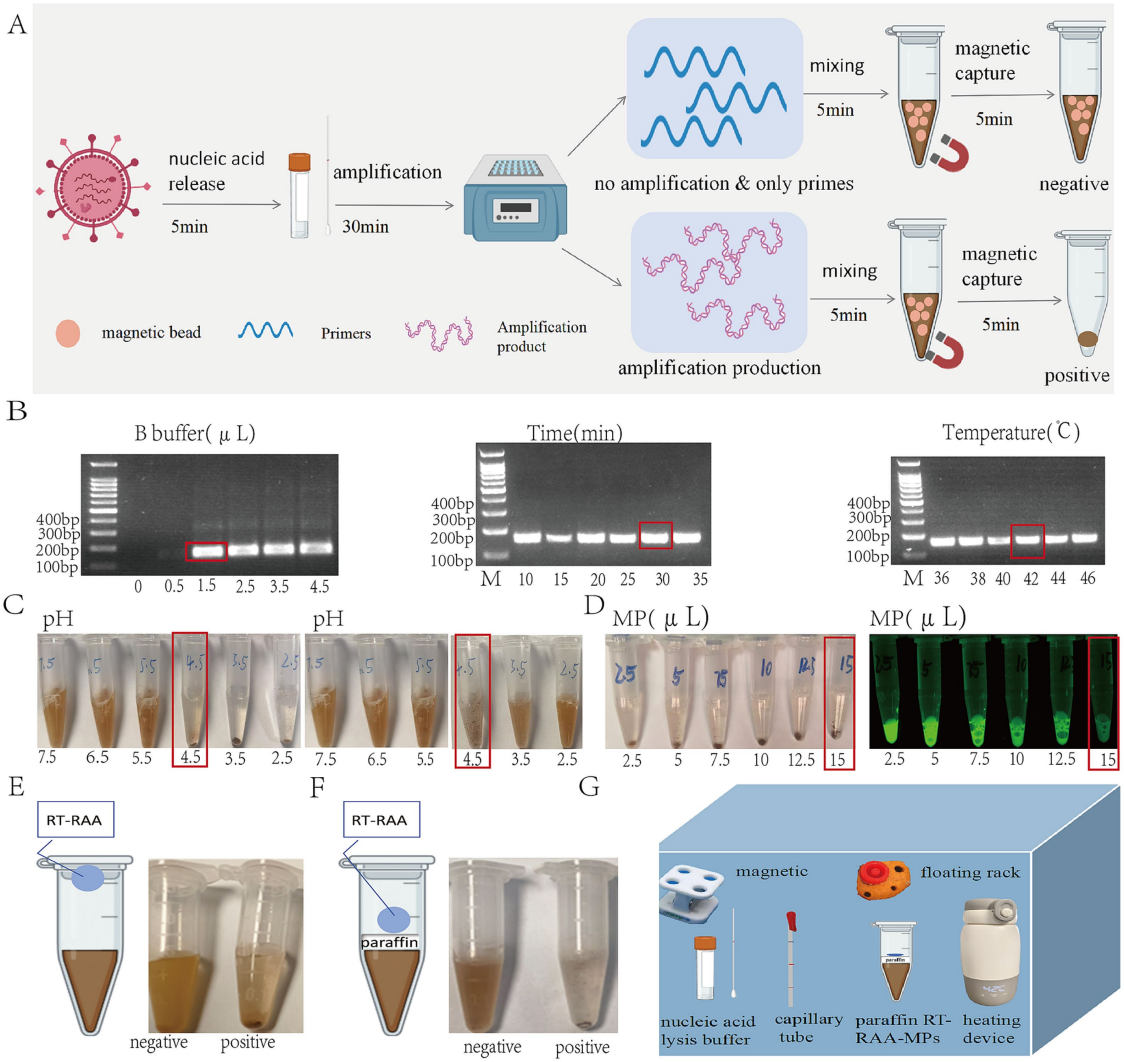
The principle of RT-RAA-MPs and optimization of RT-RAA-MPs reaction conditions. **(A)** The principle of RT-RAA-MPs. **(B)** Optimization of RT-RAA extension conditions; the red bar on the right indicates the optimal condition. **(C)** Optimization of the pH reaction conditions for RT-RAA-MPs: the left shows the RT-RAA-MPs results obtained with different pH buffers, and the right shows the results after pipette mixing. **(D)** Optimization of the magnetic bead amount for RT-RAA-MPs: the left displays the RT-RAA-MPs results with varying amounts of magnetic beads, and the right presents the bead pellet under UV illumination. **(E)** The one-tube RT-RAA-MPs. **(F)** The paraffin one-tube RT-RAA-MPs. **(G)** Equipment and reagent required for paraffin one-tube RT-RAA-MPs.

In an acidic environment, the negatively charged phosphate backbone of the long-chain DNA amplification products is partially neutralized, rendering the molecules nearly electroneutral and exposing hydrophobic bases. These hydrophobic segments simultaneously adsorb to multiple carboxyl-coated magnetic beads via hydrophobic interactions, weaving the beads into a three-dimensional network through a “hydrophobic-bridging” dual effect. This process rapidly forms visible flocculation aggregates. In contrast, in target-free systems, the magnetic beads retain their net negative charge and repel each other. When adsorbed onto a magnetic stand, the resulting precipitate is easily redispersed, and the solution remains turbid (Figure 2A).

#### 3.2.2 Optimization of RT-RAA-MPs

We optimized key parameters of the RT-RAA-MPs system to maximize performance. For RT-RAA amplification, optimal conditions were 1.5 μL of B buffer, 30 min at 42 °C (Figure 2B). For the flocculation step, pH and magnetic bead volume were systematically tested. Precipitation formed at pH 4.5, 3.5, and 2.5, with the most stable precipitate observed at pH 4.5 after pipette resuspension (Figure 2C). Magnetic bead volumes from 2.5 to 15 μL all induced precipitation; however, 15 μL captured amplification products most efficiently, as indicated by minimal supernatant fluorescence when using FAM-labeled primers (Figure 2D). These optimized conditions ensure robust flocculation and high detection sensitivity.

#### 3.2.3 Establishment of the RT-RAA-MPs system

To simplify operations and reduce aerosol contamination, a single-tube RT-RAA-MPs format was developed. The amplification mix was placed in the tube cap and the magnetic bead buffer at the bottom; centrifugation initiated binding post-reaction (Figure 2E). A paraffin layer was added to separate the components for stable storage, creating a paraffin-encapsulated system (Figure 2F). For field use, a portable setup was devised using lyophilized RT-RAA reagents, an acid-releasing agent, a capillary sampler, a magnetic stand, and an insulated cup to maintain temperature (Figure 2G). This system enables equipment-free, on-site detection.

#### 3.2.4 Performance and clinical validation of RT-RAA-MPs

**Figure 3:**
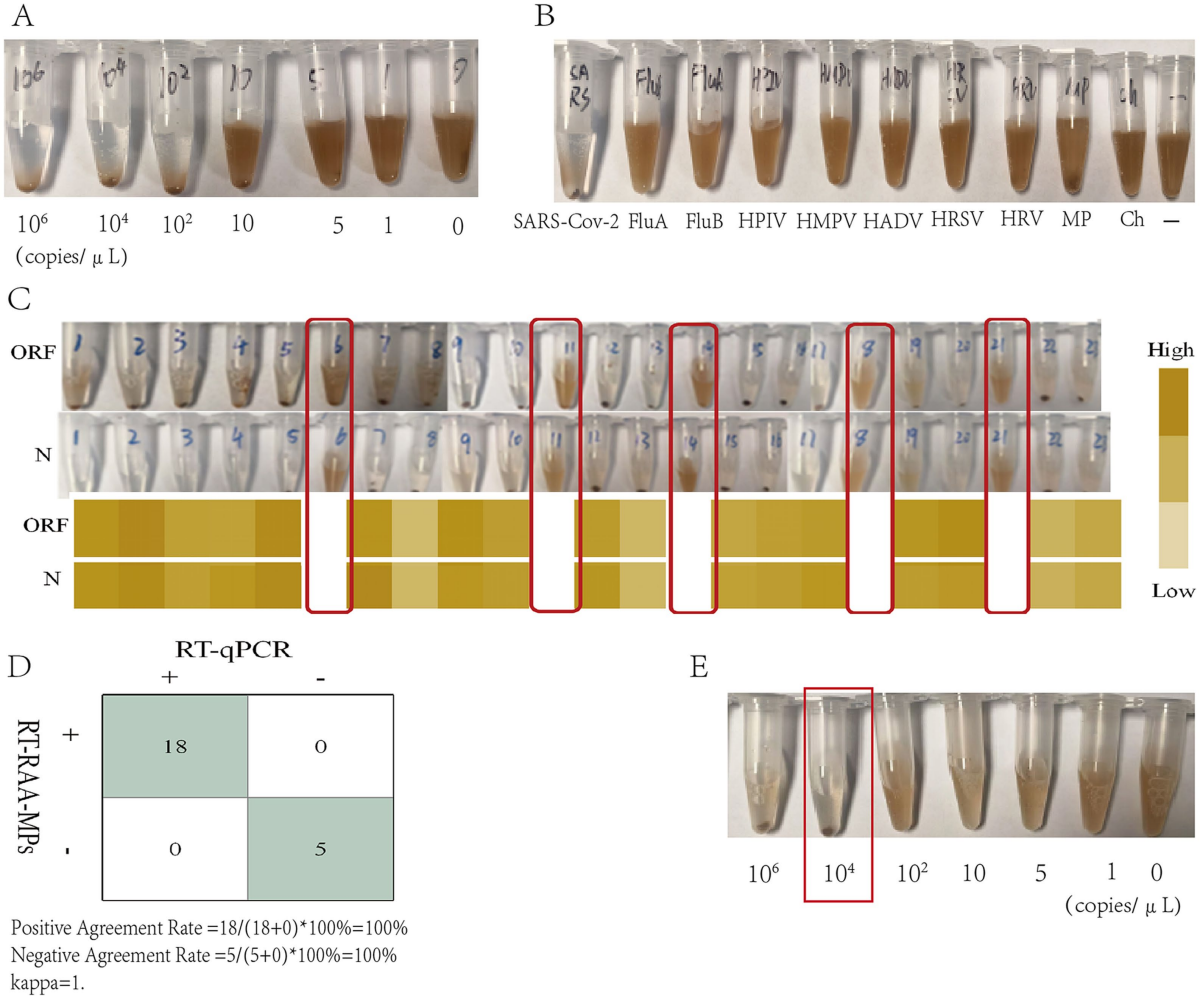
Performance validation and clinical validation of RT-RAA-MPs: **(A)** Sensitivity experiments of RT-RAA-MPs showed that no results could be detected at a concentration of 10^2^ copies/μL. **(B)** Specificity experiments of RT-RAA-MPs. **(C)** Comparison of RT-qPCR CT values and RT-RAA-MPs results for 23 clinical samples, with samples 6, 14, 18, and 21 being negative. **(D)** Statistics of RT-RAA-MPs detection results from 23 clinical samples. **(E)** The simulated water contamination RT-RAA-MPs results showed a negative outcome at a concentration of 10^4^copies/μL.

The analytical performance of RT-RAA-MPs was rigorously evaluated. Sensitivity testing with serially diluted pseudovirus showed a detection limit of 10^2^ copies/μL (3/3 at 10^2^ copies/μL, 0/3 at 10 copies/μL) (Figures 3A–B,E). Clinical Validated with 23 clinical samples and using RT-qPCR as the reference method, the performance metrics are as follows: sensitivity 100% (95% CI: 0.82–1), specificity 100% (95% CI: 0.48–1), positive predictive value 100% (95% CI, 0.82–1), negative predictive value 100% (95% CI, 0.48–1), overall percent agreement 100% (95% CI, 0.85–1), and a Kappa value of 1.00 (Figures 3C–D).

### 3.3 Establishment and performance evaluation of the RT-RAA-F-PfAgo

#### 3.3.1 Principle of the RT-RAA-F-PfAgo

**Figure 4:**
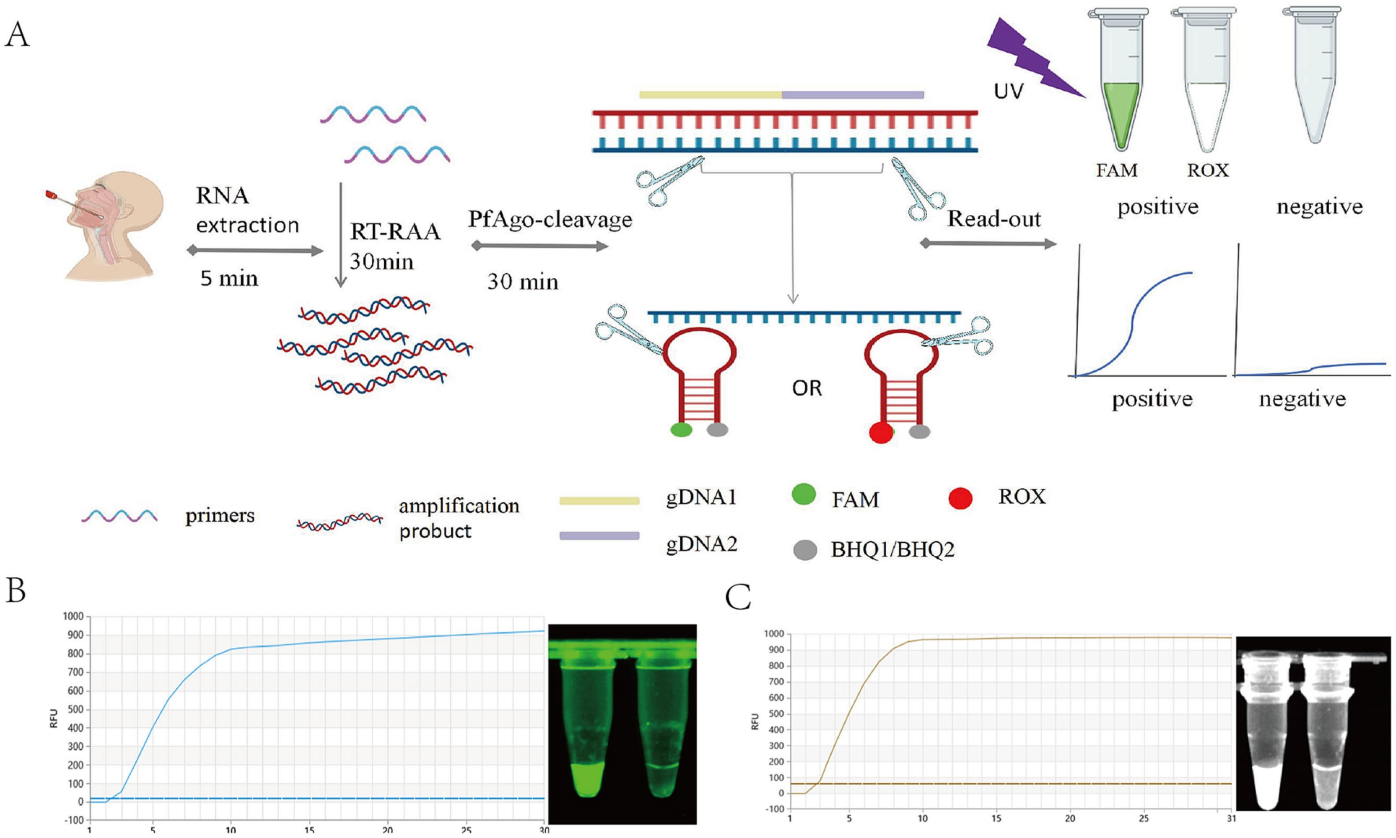
The detection principle of RT-RAA-PfAgo. **(A)** Schematic of the SARS-CoV-2 RT-RAA-F-PfAgo workflow. **(B)** The N gene RT-RAA-F-PfAgo detection. **(C)** The ORF gene RT-RAA-F-PfAgo detection.

The PfAgo protein is a DNA-guided endonuclease derived from the thermophilic archaeon *Pyrococcus furiosus*, belonging to the prokaryotic Argonaute (PfAgo) family. It utilizes 5′-phosphorylated DNA (gDNA) as a guide strand under high-temperature conditions to specifically cleave single-stranded targets. The cleavage occurs between the 10th and 11th nucleotides of the guide strand, generating a 16-nt oligonucleotide single strand. In pathogen detection, this fragment can further serve as a S-gDNA for subsequent cleavage. It complements engineered probes and guides the PfAgo protein to cleave the probes. In the RT-RAA-F-PfAgo system, cleavage of the fluorescent probe separates the fluorophore from the quencher, resulting in fluorescence emission (Figure 4A).

#### 3.3.2 Establishment of the RT-RAA-F-PfAgo

The RT-RAA-F-PfAgo assay combines RT-RAA amplification with PfAgo-mediated probe cleavage. After amplification, gDNA guides PfAgo to cleave the target, releasing S-gDNA, which then directs cleavage of dual-labeled probes (N-P-F: FAM/BHQ1; ORF-P-F: ROX/BHQ2), generating fluorescence. The reaction at 95 °C for 30 min in a qPCR instrument allowed real-time fluorescence monitoring and endpoint visualization under UV light (Figures 4B,C). This setup provides a flexible platform for fluorescence-based nucleic acid detection.

#### 3.3.3 Optimization of RT-RAA-F-PfAgo

**Figure 5:**
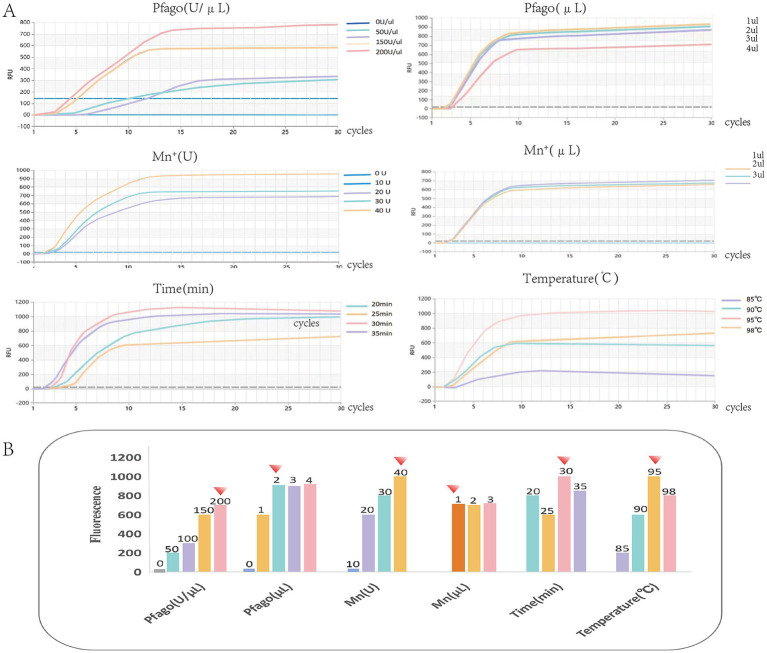
Optimization of the RT-RAA-F-PfAgo detection system. **(A)** Fluorescence curves under different conditions of Mn^+^, PfAgo, time, and temperature. **(B)** Statistical comparison of fluorescence values under different conditions of time, temperature, Mn^+^ concentration, and PfAgo concentration.

Key reaction parameters were optimized to enhance detection performance. Mn^2+^ and PfAgo protein concentrations were screened, with optimal activity at 40 U and 200 U/μL, respectively. The final 25 μL system used 1 μL of 40 U Mn^2+^ and 2 μL of 200 U/μL PfAgo. Reaction time and temperature were also tested; 30 min and 95 °C yielded the strongest and most stable fluorescence signals (Figures 5A,B). These conditions ensure efficient cleavage and high signal-to-noise ratio.

#### 3.3.4 Establishment of the RT-RAA-dF-PfAgo

**Figure 6:**
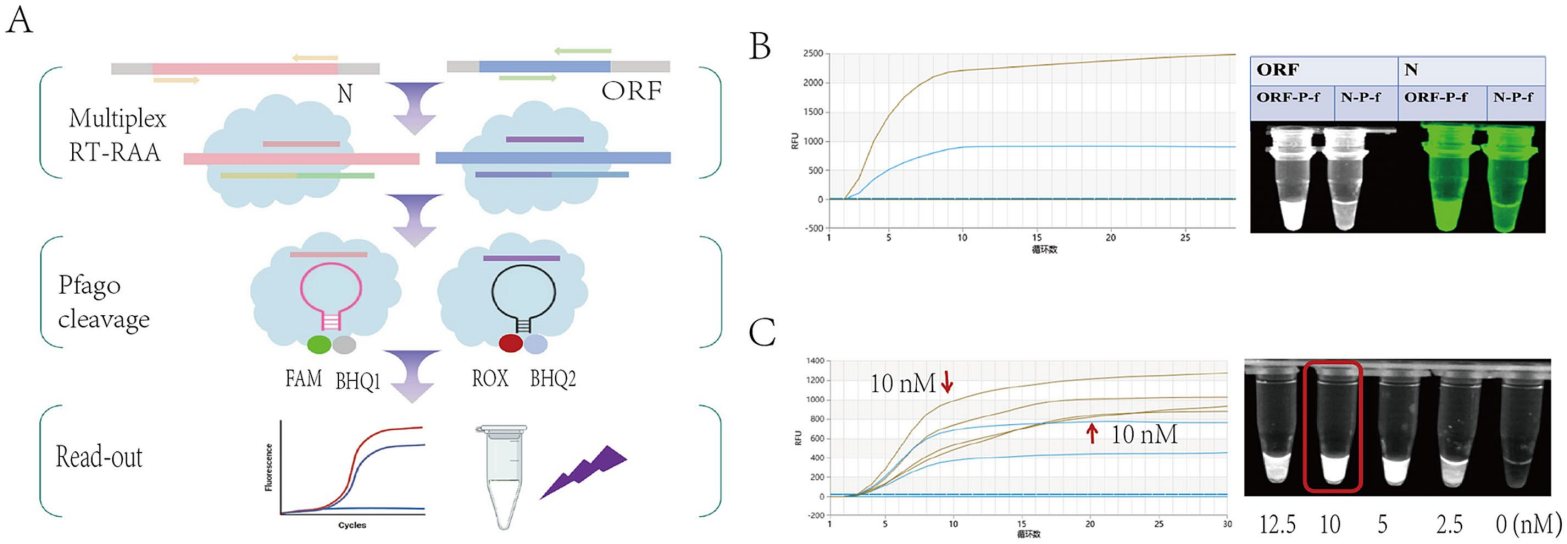
Establishment of RT-RAA-dF-PfAgo detection system. **(A)** The principle of the multiple-target detection system. **(B)** Cross-validation of probes for the ORF gene and the N gene. **(C)** Exploring the optimal probe amount.

To improve detection specificity, a single-tube dual-target fluorescence system (RT-RAA-dF-PfAgo) was established. This system simultaneously amplifies ORF and N genes and detects them using respective probes. Specificity was confirmed by observing no cross-reactivity between ORF and N probes (Figures 6A,B). Probe concentration was optimized, with 10 μL yielding maximum fluorescence intensity (Figure 6C). The RT-RAA-dF-PfAgo system enables specific and simultaneous detection of two viral targets.

#### 3.3.5 Performance and clinical validation of the RT-RAA-dF-PfAgo

**Figure 7:**
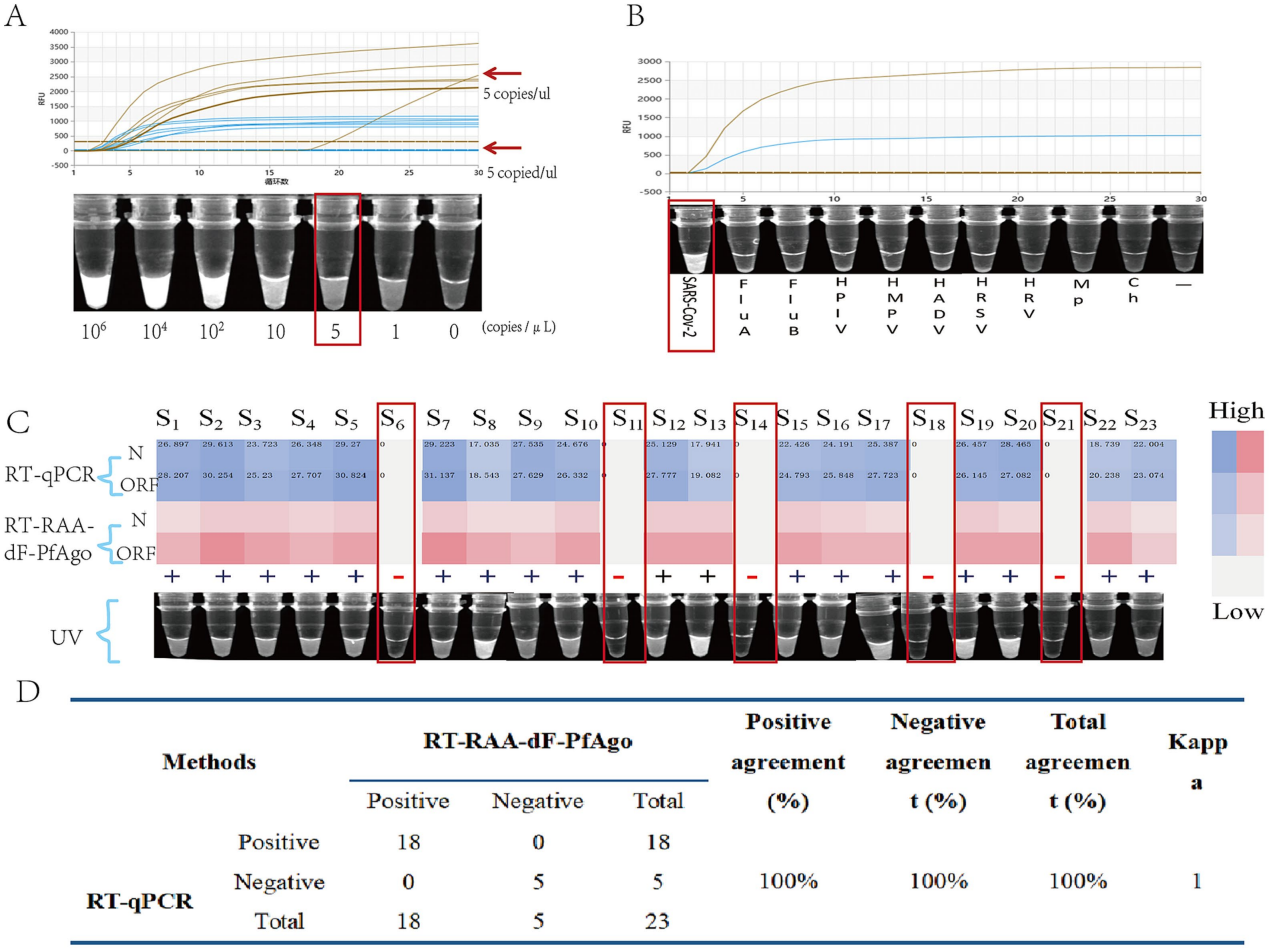
Performance validation and clinical validation of RT-RAA-dF-PfAgo: **(A)** Sensitivity experiments of RT-RAA-dF-PfAgo showed that no results could be detected at a concentration of 5 copies/μL. **(B)** Specificity experiments of RT-RAA-dF-PfAgo. **(C)** Comparison of RT-qPCR CT values and RT-RAA-dF-PfAgo results for 23 clinical samples, with samples 6, 14, 18, and 21 being negative. **(D)** Statistics of RT-RAA-dF-PfAgo detection results from 23 clinical samples.

The RT-RAA-dF-PfAgo system exhibited high sensitivity, detecting down to 5 copies/μL of pseudovirus (3/3 at 5 copies/μL, 0/3 at 1 copy/μL) (Figure 7A). It showed no cross-reactivity with other respiratory pathogens (Figure 7B). Clinical Validated with 23 clinical samples and using RT-qPCR as the reference method, the performance metrics are as follows: sensitivity 100% (95% CI: 0.82–1), specificity 100% (95% CI: 0.48–1), positive predictive value 100% (95% CI: 0.82–1), negative predictive value 100% (95% CI: 0.48–1), overall percent agreement 100% (95% CI: 0.85–1), and a Kappa value of 1.00 (Figures 7C–D).

### 3.4 Establishment and performance evaluation of the RT-RAA-L-PfAgo

#### 3.4.1 Establishment of the RT-RAA-L-PfAgo

**Figure 8:**
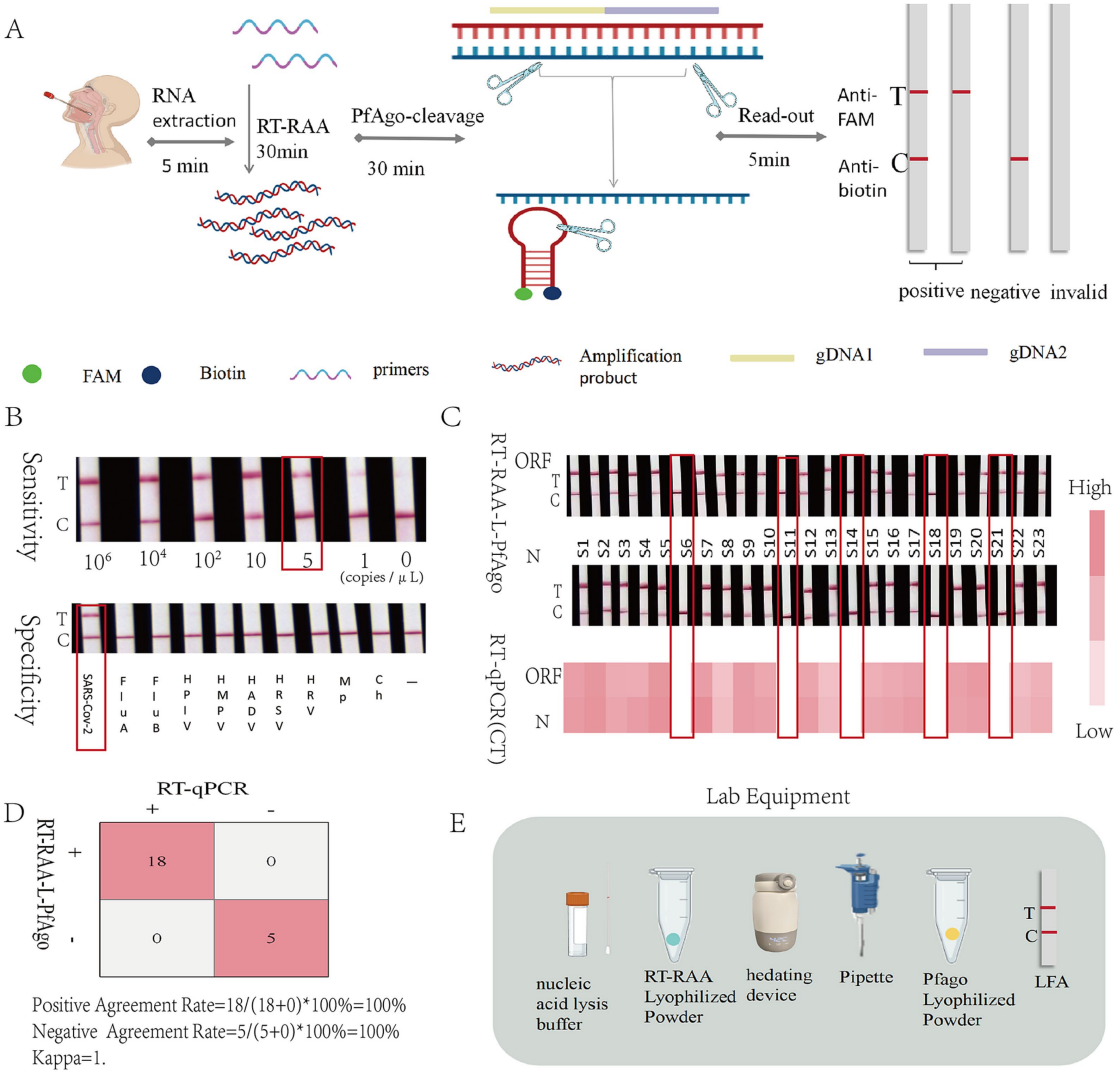
Establishment of RT-RAA-L-PfAgo detection system and performance validation. **(A)** The principle of the RT-RAA-L-PfAgo detection system. **(B)** Sensitivity and specificity experiments of RT-RAA-L-PfAgo. For the sensitivity experiment, results showed that no results could be detected at a concentration of 5 copies/μL. **(C)** Comparison of RT-qPCR CT values and RT-RAA-L-PfAgo results for 23 clinical samples, with samples 6, 14, 18, and 21 being negative. **(D)** Statistics of RT-RAA-L-PfAgo detection results from 23 clinical samples. **(E)** Equipment and reagent required for RT-RAA-L-PfAgo.

For rapid and instrument-free detection, a lateral flow strip-based method was developed. Dual-labeled probes (FAM and biotin) are cleaved by PfAgo in the presence of target, separating FAM from biotin. Cleaved FAM binds anti-FAM antibody on the test line (T), while biotin binds streptavidin on the control line (C), producing both T and C lines for positive results; intact probes yield only a C line (Figure 8A). Lyophilization of RT-RAA and PfAgo reagents facilitated storage and field use with minimal equipment (Figure 8E). This approach provides a practical solution for point-of-care testing.

#### 3.4.2 Performance and clinical validation of the RT-RAA-L-PfAgo

The RT-RAA-L-PfAgo assay demonstrated a detection sensitivity of 5 copies/μL (3/3 at 5 copies/μL, 1/3 at 1 copy/μL) (Figure 8B) and high specificity, with no cross-reactivity against other respiratory viruses (Figure 8C). Clinical Validated with 23 clinical samples and using RT-qPCR as the reference method, the performance metrics are as follows: sensitivity 100% (95% CI: 0.82–1), specificity 100% (95% CI: 0.48–1), positive predictive value 100% (95% CI: 0.82–1), negative predictive value 100% (95% CI: 0.48–1), overall percent agreement 100% (95% CI: 0.85–1), and a Kappa value of 1.00 (Figure 8D).

The performance verification results and characteristics of RT-RAA-MPs, RT-RAA-dF-PfAgo, and RT-RAA-L-PfAgo are shown in Table 2.

**Table 2:** Characteristic of RT-RAA-MPs and RT-RAA-PfAgo.

Characteristic	RT-RAA-MPs	RT-RAA-dF-PfAgo	RT-RAA-L-PfAgo
Sensitivity	10^2^ copies/μL	5 copies/μL	5 copies/μL
Specificity	Medium	High	High
Positive agreement	100%	100%	100%
Negative agreement	100%	100%	100%
Kappa	1	1	1
Time	40 min	65 min	60 min
Apparatus	Adjustable and controlled thermos	Fluorometer or qPCR machine	Adjustable and controlled thermos
Advantage	Low-cost, rapid, naked-eye detection, and independent equipment	High sensitivity and specificity	High sensitivity with minimal instrumentation
Limitation	Medium sensitivity specificity and single-target detection	Requires sophisticated instrumentation	Single-target detection
Applicable scenarios	Home testing, low-resource settings, POCT	Clinical laboratories	All level-hospital setting

It should be noted that the evaluation levels of sensitivity and specificity presented in the table are based on a relative comparison among the three detection techniques.

## 4 Discussion

The SARS-Cov-2 pandemic has relentlessly exposed the vulnerabilities of global public health systems, particularly the critical limitations of conventional diagnostic methodologies in facing rapid, large-scale, and decentralized testing demands (Ferencova et al., 2024; Mercer and Salit, 2021). Centralized laboratory-based techniques such as RT-qPCR and next-generation sequencing, despite their high accuracy, are constrained by substantial infrastructure requirements, expensive instrumentation, and operational complexity, rendering them poorly suited for resource-limited environments, POCT scenarios, or home-use settings (Zhao et al., 2024a; Alhamid et al., 2022). This glaring diagnostic gap has necessitated the development of innovative testing strategies that are not only accurate and rapid but also economically feasible and instrument-light. In direct response to this unmet need, our study designed and rigorously validated two novel RT-RAA-based detection platforms—a one-pot magnetic bead flocculation (MPs) system and a programmable PfAgo-based platform—which collectively establish a versatile, robust, and scenario-adaptive framework for SARS-CoV-2 detection, effectively bridging the technological divide between centralized confirmation and field-based screening.

We first established the one-pot RT-RAA-MPs system with the explicit aim of delivering a truly equipment-free, visually interpretable molecular assay suitable for home testing, low-resource settings, and environmental surveillance. Through systematic optimization, we identified pH 4.5 as optimal for rapid flocculation, achieving complete precipitation within 10 min. This system consistently detected SARS-CoV-2 pseudovirus down to 10^2^ copies/μL and demonstrated 100% clinical agreement with RT-qPCR across 23 patient samples. Crucially, its integrated, paraffin-sealed single-tube design eliminated post-amplification handling. When tested with simulated environmental water samples, the assay achieved a detection limit of 10^4^ copies/μL; nevertheless, sensitivity remains suboptimal due to the absence of RNA enrichment and potential inhibitors present in environmental matrices. By combining lyophilized reagents with a portable insulated cup and capillary-based operation, the system enables instrument-free, sub-40 min detection without electricity or technical expertise, thereby positioning the RT-RAA-MPs system as a transformative tool for empowering testing at the community, home, and environmental levels.

POCT detection technologies include antigen–antibody colloidal gold assays and nucleic acid-based POCT methods. Although traditional antigen-based POCT products can complete detection within 15–30 min with minimal equipment requirements, their analytical sensitivity is generally low, leading to a high false-negative rate for samples with low viral loads. Nucleic acid detection technologies have gained considerable attention due to their high sensitivity and specificity. Currently, commercial nucleic acid POCT systems include the Cepheid GeneXpert, Abbott ID NOW, and BioFire FilmArray systems (Song et al., 2021). Compared to conventional PCR techniques, the detection time for these systems is significantly reduced to approximately 30 min, and researchers have focused on minimizing equipment complexity and size. However, this has also increased equipment costs, and the newly developed devices are not widely available on the market. In contrast, the RT-RAA-MPs system developed in this study achieves a detection limit of 10^2^ copies/μL, with a total detection time of 40 min. The equipment cost is relatively low, and the required devices are commonly available and easily accessible.

To address scenarios requiring higher order specificity and flexible readout capabilities, we developed the RT-RAA-PfAgo platform, which integrates the programmability of PfAgo with RT-RAA. The fluorescence-based version, including both single-target (RT-RAA-F-PfAgo) and dual-target (RT-RAA-dF-PfAgo) formats, was systematically optimized to function with 40 U Mn^2+^and 200 U/μL PfAgo at 95 °C for 30 min, achieving a remarkable detection limit of 5 copies/μL. The dual-target system, employing 10 μM probes for both ORF and N genes, showed no cross-reactivity and exhibited perfect clinical concordance (Kappa = 1.00) with RT-qPCR, significantly enhancing diagnostic reliability through multi-gene verification. Offering quantitative results and a broad dynamic range (10^1^–10^6^ copies/μL), this platform serves as an ideal solution for clinical laboratories requiring high sensitivity and specificity without sacrificing throughput or quantitative capability.

For demanding rapid visual interpretation, we further engineered the RT-RAA-L-PfAgo system, which transduces PfAgo-mediated cleavage into a lateral flow readout. Utilizing dual-labeled probes, the system produces clear visual results within 5–10 min post-reaction, while retaining the high sensitivity (5 copies/μL) and exceptional specificity (no cross-reactivity across nine respiratory pathogens) intrinsic to the PfAgo mechanism. Clinical validation again confirmed 100% agreement with RT-qPCR across the same 23 samples, supporting its use in settings such as pharmacies, community clinics, or field stations where speed, simplicity, and accuracy are paramount. This lateral flow format effectively extends the utility of the PfAgo platform to users who require definitive visual results without any supporting instrumentation.

When evaluated collectively, the RT-RAA-MPs and RT-RAA-PfAgo platforms offer complementary advantages across a spectrum of real-world application scenarios. The MPs system operates with minimal equipment (<$125–340 setup) and reduced reagent costs (about two-fifths of commercial RT-qPCR kits), making it uniquely suited for home use, resource-poor regions, and environmental monitoring networks. In contrast, the PfAgo-based systems provide a tiered detection strategy: the fluorescence versions leverage existing qPCR instruments for quantitative, lab-based confirmation, while the lateral flow version delivers rapid, equipment-free readouts for decentralized screening. Together, they present a scalable and cost-effective alternative to RT-qPCR and NGS—technologies that remain hampered by high capital costs, extended turnaround times, and complex operational demands—there substantially expanding access to precision molecular diagnostics.

It is important to acknowledge certain limitations of the current study. Although the clinical validation yielded excellent agreement with RT-qPCR, the sample size remains moderate, warranting future multi-center studies with larger and more diverse cohorts to reinforce generalization. It should be noted that the specificity validation in this study only covered nine common respiratory pathogens. Limited by the variety of samples, this scope is insufficient to fully reflect the complexity of real clinical specimens, and future studies should include a broader range of pathogens for validation. Due to laboratory safety considerations, this study utilized pseudoviruses instead of live viruses for analytical sensitivity testing, which may represent a limitation. Although the effectiveness of the detection technique described in this study has been demonstrated through clinical sample validation, pseudovirus reagents cannot fully replace actual clinical specimens. Future research will incorporate more clinical samples and live viruses to further validate the clinical applicability of this detection technique. The long-term stability of lyophilized reagents under variable storage conditions also requires further evaluation to ensure consistent performance in real-world distribution channels. Moreover, continuous monitoring and potential refinement of primer and gDNA designs will be essential to maintain detection efficacy against emerging viral variants. And, in future research, we will also consider developing it into a quantitative analytical method, and explore ways to enhance the objectivity and comparability of results by introducing semi-quantitative or quantitative auxiliary indicators (such as intra-/inter-assay CV data), combining with other analytical techniques, and optimizing data processing workflows.

In conclusion, this work introduces two robust, complementary diagnostic platforms that effectively address the pressing need for accessible, accurate, and adaptable SARS-CoV-2 testing. The one-pot RT-RAA-MPs system breaks new ground in instrument-free visual molecular detection, while the RT-RAA-PfAgo platform offers a highly specific, multi-format detection strategy suitable for both quantitative laboratory analysis and rapid field testing. By demonstrating high analytical sensitivity, specificity, and clinical accuracy—coupled with significant reductions in cost, complexity, and instrument dependency—these technologies provide a scalable and versatile foundation for strengthening diagnostic resilience at all healthcare levels, thereby enhancing global preparedness for current and future infectious disease threats.

## Data Availability

The datasets presented in this study can be found in online repositories. The names of the repository/repositories and accession number(s) can be found in the article/supplementary material.
